# Transcriptome comparison and gene coexpression network analysis provide a systems view of citrus response to ‘*Candidatus* Liberibacter asiaticus’ infection

**DOI:** 10.1186/1471-2164-14-27

**Published:** 2013-01-16

**Authors:** Zhi-Liang Zheng, Yihong Zhao

**Affiliations:** 1 Plant Nutrient Signaling and Fruit Quality Improvement Laboratory, Citrus Research Institute & College of Horticulture and Landscape Architecture, Southwest University, Beibei, Chongqing, 400712, China; 2 Department of Psychiatry, Columbia University, New York, NY, 10032, USA

## Abstract

**Background:**

Huanglongbing (HLB) is arguably the most destructive disease for the citrus industry. HLB is caused by infection of the bacterium, *Candidatus* Liberibacter spp. Several citrus GeneChip studies have revealed thousands of genes that are up- or down-regulated by infection with *Ca.* Liberibacter asiaticus. However, whether and how these host genes act to protect against HLB remains poorly understood.

**Results:**

As a first step towards a mechanistic view of citrus in response to the HLB bacterial infection, we performed a comparative transcriptome analysis and found that a total of 21 Probesets are commonly up-regulated by the HLB bacterial infection. In addition, a number of genes are likely regulated specifically at early, late or very late stages of the infection. Furthermore, using Pearson correlation coefficient-based gene coexpression analysis, we constructed a citrus HLB response network consisting of 3,507 Probesets and 56,287 interactions. Genes involved in carbohydrate and nitrogen metabolic processes, transport, defense, signaling and hormone response were overrepresented in the HLB response network and the subnetworks for these processes were constructed. Analysis of the defense and hormone response subnetworks indicates that hormone response is interconnected with defense response. In addition, mapping the commonly up-regulated HLB responsive genes into the HLB response network resulted in a core subnetwork where transport plays a key role in the citrus response to the HLB bacterial infection. Moreover, analysis of a phloem protein subnetwork indicates a role for this protein and zinc transporters or zinc-binding proteins in the citrus HLB defense response.

**Conclusion:**

Through integrating transcriptome comparison and gene coexpression network analysis, we have provided for the first time a systems view of citrus in response to the *Ca.* Liberibacter spp. infection causing HLB.

## Background

Orange juice is among the largest beverage industries in the world. Sweet orange is mainly produced in the subtropical areas in the countries of China, US and Brazil and the Mediterranean basin regions. Sweet orange (*Citrus sinensis*) belongs to the *Citrus* genus that includes several other species such as tangerine, mandarin and grapefruit. In horticultural practice, citrus is asexually propagated through grafting the scion onto the stock which is grown through the seeds. The scion has been bred for the desired traits of fruit quality while the stock is mostly selected for supporting the optimal scion growth and increased resistance to biotic and abiotic stresses.

Among the major biotic factors which frequently challenge tree growth and fruit development, Huanglongbing (HLB) or called citrus greening is one of the most destructive diseases. HLB was first reported in 1919 in southern China, and very recently it has been reported in almost all major citrus production areas [1-3]. For example, in Florida alone, HLB has caused the loss of several billion dollars since 2005 when HLB was first reported, ranging from 30–100% of loss in fruit production in citrus groves [3]. HLB is caused by infection of the bacterium, *Candidatus* Liberibacter spp., which is spread to plants via the vector Asian citrus psyllid (*Diaphorina citri*) or through grafting of a diseased shoot. The HLB bacterium has three species, *Ca.* Liberibacter asiaticus (Las), *Ca.* Liberibacter africanus (Laf) and *Ca.* Liberibacter americanus (Lam). The genome of the Las species was recently published, with a size of approximately 1.23 Mb [4]. It has been generally accepted that, after infection or inoculation, the HLB bacteria migrate through phloem and, by accumulating there, causes the formation of sieve plug [2,3,5-7]. Consequently, the transport of nutrients (such as carbohydrates and amino acids) from the source leaves to various sinks (such as young leaves, fruits and roots) are compromised or even blocked in severely infected plants, leading to the alterations in carbohydrate metabolism for metabolic flow and exhibiting such phenotypes as yellow and blotchy mottles on leaves, variegated fruits and poor root growth [6-8]. Because of the huge impact of HLB in the citrus industry, plant pathologists and horticulturists have long sought after the HLB resistance mechanism in citrus.

A recent survey suggests the existence of genetic variations among different citrus species, varieties and stocks [9]. In general, mandarin, sweet orange and grapefruit are relatively more susceptible to the HLB bacterial infection, while sour orange, lemon, lime, and citrange (a hybrid of sweet orange and the stock *C. trifoliata*) are less susceptible. This raises the possibility that HLB resistance can be achieved through genetic means. Nevertheless, breeding for the HLB resistance through crossing will be a daunting task, given the complex genetic backgrounds, the nature of asexual propagation and the relatively long juvenile period for citrus. Therefore, many researchers have turned their attentions to finding the target genes that are required or critical for the citrus host response to the HLB bacteria. Transcriptome analysis has been used as a straight forward approach to identify the genes whose expression is altered in citrus leaves in response to the HLB inoculation [5,10-12]. These studies led to the identification of several hundred or thousand genes that are up- or down-regulated by the HLB bacterial infection. The majority of these genes can be grouped into metabolism, transport and response to stimulus. However, these studies varied significantly in terms of study design (including experimental materials and infection stages) and data analysis (for example, different statistical methods and various fold-change cutoffs). Furthermore, there is a lack of comparison of the results from these different experiments. In addition, how these HLB bacterium-regulated genes are connected in a system remains unknown.

To provide a systems view of citrus response to the HLB bacterial infection, we first performed a comparative study of the previously reported transcriptome datasets. Our results show that there are 21 probe sets (representing up to 19 genes) are commonly up-regulated and a number of genes that are specific to early, late or very late stages of inoculation. Furthermore, using the Pearson correlation coefficient (Pcc)-based unweighted gene coexpression analysis, we constructed an HLB response network. This citrus gene coexpression network consists of 3,507 Probesets and 56,857 interactions. We then mapped certain categories of the HLB responsive genes to the HLB response network, resulting in the formation of several important subnetworks including metabolism, transport, signaling, defense response and hormone response. Taken together, through comparative transcriptome analysis and construction of a citrus gene coexpression analysis, we have provided a systems view of citrus response to the *Ca.* Liberibacter infection causing HLB.

## Results

### An overview of comparative analysis of HLB transcriptomes

To perform a comparative transcriptome study, we decided to use the same data pre-processing and statistical analysis methods and the same selection criteria for the identification of HLB significantly regulated genes. Two sets of the citrus Affymetrix GeneChip data derived from very recent publications [11,12] were retrieved from the NCBI Gene Expression Omnibus (GEO) database, while the data for the two earlier reports were provided by Drs. Bowman [10] and Wang [5], respectively. These four reports represent six different studies that can be used for individual comparisons, with a total of 34 arrays (see Table 1). In these studies, genome-wide gene expression was profiled from the citrus leaves inoculated by the HLB bacterium Las. However, these six studies can be categorized into three distinct HLB disease stages (see Table 1). Because the three studies used the leaf samples 30–35 weeks after inoculation, we arbitrarily called this “very late” stage by following the definition of “early” (5–9 weeks) and “late” stages (13–17 weeks) described in the first HLB transcriptome study [10].

**Table 1:** A list of citrus GeneChip studies used in this analysis

**Studies**	**Varieties**	**Weeks after inoculation**	**Ref.**	**Stages**	**No. of significantly regulated Probesets reported in this study**	
					**Up-regulated**	**Down-regulated**
1	Valencia	5–9	[10]	Early	158	62
2	Valencia	13–17	[10]	Late	394	285
3	Sweet orange	35	[5]	Very late	942	1060
4	Madam Vinous	30	[11]	Very late	1955	2125
5	Cleopatra	30–32	[12]	Very late	1110	626
6	US-897	30–32	[12]	Very late	261	39

The citrus GeneChip contains a total of 30,173 Probesets. Because the Affymetrix company has not provided a comprehensive annotation analysis for those Probesets, it is not known how many unique citrus genes are actually represented in the chip. Therefore, we decided to analyze the number of Probesets that are significantly regulated in response to HLB. The data pre-processing was described in Methods. In brief, those Probesets with the calls of present (*P*) or marginal (*M*) in at least two chips in each of the four reports were included in our analysis. For the identification of significantly regulated genes, the adjusted LPE approach was used because of its power in analyzing small samples [13]. In our analysis, a two-fold cutoff was used, resulting in various numbers of genes that were either up- or down-regulated in each of the six studies (see Table 1). The HLB regulated genes for each study were listed in Additional file 1. If the genes significantly regulated in at least one study were added together, we found that a total of 3,345 and 3,230 Probesets were up-regulated and down-regulated, respectively. These Probesets are called “HLB responsive genes” in this study (Additional file 1). The percentage of “HLB responsive genes” identified in this comparative analysis (22%) is similar to that of the bacterial pathogen responsive genes in Arabidopsis [14]. This indicates that either the disease response mechanism could be somehow conserved or these four reports have probably identified most of the HLB responsive genes in the citrus genome. Surprisingly, the study-wise comparison showed that the proportion of the significantly regulated genes overlapped in two of the six studies varied dramatically (from 0.05% to 75%) (see Additional file 2).

### Comparative studies reveal commonly regulated and stage-specifically regulated genes by HLB

Despite our finding that only a small proportion of Probesets are significantly regulated in any of two studies, we reasoned that those Probesets (and their represented genes) commonly regulated in all of the studies may represent either a common core pathway or default pathway in response to the Las infection. We first found a total of 13 Probesets that are commonly up-regulated in all of the six studies (see Table 2), representing only 0.4% (14 out of 3,426) of the HLB up-regulated genes. However, the number of Probesets significantly regulated in any of five studies increased to 42. It is possible that in the absence of the HLB bacterial challenge some of the HLB up-regulated genes already had higher transcript levels in the relatively resistant germplasm US-897 compared to the relatively susceptible mandarin Cleopatra and thus they were not up-regulated any more in US-897 in response to the Las infection; however, they could be significantly regulated in all other four studies. We did identify a total of eight Probesets for this type of expression pattern and consequently they were also added to the list of the HLB commonly regulated genes (see Table 2). Surprisingly, there was no Probeset commonly down-regulated in all of the six studies and only one Probeset that is significantly down-regulated in five studies. This Probeset, Cit.18719.1.S1_at, is annotated to encode a gene similar to Arabidopsis AT5G18600-encoded glutaredoxin family protein involved in cell redox homeostasis. Gene Ontology (GO) analysis of the subset of 21 commonly up-regulated Probesets indicates that metabolism (5 Probesets), transport (5 Probesets), hormone responses (3 Probesets) and unknown processes (7 Probesets) are the largest groups. The three Probesets representing the genes involved in hormone response indicate that gibberelic acid (GA), abscisic acid (ABA), auxin, ethylene and jasmonic acid (JA) may have certain role in mediating the citrus response to HLB. Interestingly, three Probesets (Cit.30071.1.S1_at, Cit.33707.1.S1_at, and Cit.3534.1.S1_s_at) belonging to the category of “unknown process” might also be involved in ethylene response as they exhibit the highest homology to genes that are associated with ethylene response using the manual BLAST search. In addition, there is a transcription factor gene represented by the Probeset, Cit.12214.1.S1_s_at (an NAC domain containing protein called NAC096 in Arabidopsis) and another putative RAP2.4-like ethylene transcription factor represented by Cit.3534.1.S1_s_at. Taken together, the existence of these commonly up-regulated genes strongly indicates that metabolism, transport, hormone response and transcriptional regulation play a critical role and may define the default or basal pathways in citrus during the whole process of the Las infection.

**Table 2:** A list of commonly up-regulated HLB responsive genes in various studies

**Probeset ID**	**AtGID**	**Arabidopsis gene annotation**	**GO term**
Cit.10032.1.S1_x_at	AT1G75750	GA-responsive GAST1 homolog; BR, GA and ABA responsive expression; located in cell wall; GASA1 (GAST1 PROTEIN HOMOLOG 1)	Response to stimulus
Cit.12214.1.S1_s_at	AT5G46590	NAC096 (Arabidopsis NAC domain containing protein 96); transcription factor	Transcription
Cit.13437.1.S1_s_at	AT4G39210	APL3 (sugar-inducible); glucose-1- phosphate adenylyltransferase	Carbohydrate metabolic process
Cit.17561.1.S1_s_at	AT5G05365	Metal ion binding	Transport
Cit.19674.1.S1_s_at	AT3G45140	LOX2 (LIPOXYGENASE 2); lipoxygenase; response to JA, bacterium, fungus and wounding; JA biosynthetic process	Response to stimulus
Cit.25840.1.S1_s_at	AT1G17840	WBC11 (WHITE-BROWN COMPLEX HOMOLOG PROTEIN 11); ATPase, coupled to transmembrane movement of substances / fatty acid transporter; response to stress and ABA	Transport; Response to stimulus
Cit.30071.1.S1_at	AT5G02580	Unknown protein (auxin-ethylene interaction, gemivirus induced)	Unknown process
Cit.33707.1.S1_at	AT4G27900	CCT motif family protein (ethylene, gemivirus-induced)	Unknown process
Cit.7635.1.S1_at	AT3G15650	Phospholipase/carboxylesterase family protein	Other metabolic process
Cit.5367.1.S1_at		Transcript Assignment: DN622378; Human protein tyrosine phosphatase type IVA	Unknown process
Cit.3534.1.S1_s_at		Grape RAP2-4-like ethylene transcription factor	Unknown process
Cit.29171.1.S1_at		UCRPT02_65A04_b *Poncirus trifoliata* Roots with Iron Deficiency cDNA clone	Unknown process
Cit.19313.1.S1_at		Transcript Assignment: CX308038; Hypothetical protein	Unknown process
Cit.11459.1.S1_s_at	AT1G05300	ZIP5; cation/metal ion transmembrane transporter	Transport
Cit.11460.1.S1_at	AT1G05300	ZIP5; cation/metal ion transmembrane transporter	Transport
Cit.12172.1.S1_at	AT4G35160	O-methyltransferase family 2 protein	Other metabolic process
Cit.15630.1.S1_at	AT4G33040	Glutaredoxin family protein	Oxidation-reduction process
Cit.17155.1.S1_at	AT1G02850	BGLU11 (BETA GLUCOSIDASE 11); catalytic/ cation binding / hydrolase, hydrolyzing O-glycosyl compounds	Carbohydrate metabolic process
Cit.17155.1.S1_s_at	AT1G02850	BGLU11 (BETA GLUCOSIDASE 11); catalytic/ cation binding / hydrolase, hydrolyzing O-glycosyl compounds	Carbohydrate metabolic process
Cit.3171.1.S1_x_at	AT1G18880	Proton-dependent oligopeptide transport (POT) family protein	Transport
Cit.23598.1.S1_s_at			Unknown process

The first section contains 13 ProbeSets up-regulated in all six studies. The second section includes eight Probesets up-regulated in five studies (except Study 6, see Table 1) but their expression was higher in US-897 than in Cleopatra. AtGID, Arabidopsis gene ID.

In contrast to the commonly regulated genes in HLB response, we found various numbers of stage-specifically regulated genes as this group of genes were only regulated at a particular stage. There are 27 and 7 Probesets that are respectively up-regulated and down-regulated only at early stage, and thus they belong to the group of early stage-specific genes (Table 3). In this early stage-specific group, the genes encoding components involved in cell wall metabolism and transcription are of particular interest. First, there are six Probesets that could potentially represent the genes involved in cell wall biogenesis or property (Cit.17310.1.S1_s_at, Cit.17450.1.S1_s_at, Cit.23171.1.S1_x_at, Cit.33335.1.S1_s_at, Cit.35345.1.S1_s_at, and Cit.30425.1.S1_x_at). Second, five Probesets represent the transcription factors homologous to Arabidopsis ERF5 (Ethylene responsive element-binding factor), ATAF1 and ARR9 (a two-component response regulator). In addition, Cit.16537.1.S1_at represents a GCN5-related N-acetyltransferase family protein, which might be involved in global transcriptional control through chromatin remodeling. This result implies that transcriptional control and cell wall property regulation are among the early events in citrus in response to the HLB bacterial attack.

**Table 3:** A list of early stage-specific HLB responsive genes

**Probeset ID**	**FC**	**AtGID**	**Arabidopsis gene annotation**
***Up-regulated***			
Cit.12388.1.S1_at	2.1	AT2G40095	unknown protein
Cit.13969.1.S1_s_at	2.1	AT5G52640	ATHSP90.1 (HEAT SHOCK PROTEIN 90.1); ATP binding / unfolded protein binding
Cit.15958.1.S1_at	2.3	AT2G26560	PLA2A (PHOSPHOLIPASE A 2A); lipase/ nutrient reservoir
Cit.16156.1.S1_at	2.7	AT5G41330	potassium channel tetramerisation domain-containing protein
Cit.16537.1.S1_at	2.4	AT2G32030	GCN5-related N-acetyltransferase (GT) family protein
Cit.17187.1.S1_at	2.7	AT1G05575	unknown protein
Cit.17310.1.S1_s_at	2.2	AT1G32170	XTR4 (XYLOGLUCAN ENDOTRANSGLYCOSYLASE 4); hydrolase, acting on glycosyl bonds
Cit.17450.1.S1_s_at	2.7	AT2G46330	AGP16 (ARABINOGALACTAN PROTEIN 16)
Cit.18156.1.S1_at	2.2	AT3G02840	Immediate-early fungal elicitor family protein
Cit.18603.1.S1_at	2.5		
Cit.18603.1.S1_s_at	2.1		
Cit.1968.1.S1_s_at	2.4	AT1G61680	TPS14 (TERPENE SYNTHASE 14); S-lilool synthase
Cit.23171.1.S1_x_at	2.0	AT3G22120	CWLP (CELL WALL-PLASMA MEMBRANE LINKER PROTEIN); lipid binding
Cit.24979.1.S1_at	2.5		
Cit.29504.1.S1_s_at	3.8	AT4G20820	FAD-binding domain-containing protein
Cit.29533.1.S1_s_at	2.6	AT5G47230	ERF5 (ETHYLENE RESPONSIVE ELEMENT BINDING FACTOR 5); D binding / transcription activator/ transcription factor
Cit.31964.1.S1_at	2.2		
Cit.33263.1.S1_at	2.3		
Cit.33335.1.S1_s_at	2.4	AT3G22120	CWLP (CELL WALL-PLASMA MEMBRANE LINKER PROTEIN); lipid binding
Cit.34429.1.S1_s_at	2.8	AT5G08350	GRAM domain-containing protein / ABA-responsive protein-related
Cit.3449.1.S1_at	2.3	AT1G01720	ATAF1; transcription activator/ transcription factor
Cit.3449.1.S1_s_at	2.4	AT1G01720	ATAF1; transcription activator/ transcription factor
Cit.3450.1.S1_at	2.5	AT1G01720	ATAF1; transcription activator/ transcription factor
Cit.35345.1.S1_s_at	2.1	AT3G28340	GATL10 (Galacturonosyltransferase-like 10); polygalacturote 4-alpha-galacturonosyltransferase/ transferase, transferring hexo
Cit.4259.1.S1_at	2.2	AT1G14870	Response to oxidative stress; LOCATED IN: plasma membrane
Cit.4690.1.S1_at	2.8	AT5G07990	TT7 (TRANSPARENT TESTA 7); flavonoid 3'-monooxygese/ oxygen binding
Cit.9944.1.S1_x_at	2.1	AT4G37990	ELI3-2 (ELICITOR-ACTIVATED GENE 3–2); aryl-alcohol dehydrogese/ mannitol dehydrogese
***Down-regulated***			
Cit.16792.1.S1_at	2.1	AT3G57040	ARR9 (RESPONSE REGULATOR 9); transcription regulator/two-component response regulator
Cit.18730.1.S1_at	2.1		
Cit.23498.1.S1_s_at	2.2	AT3G12750	ZIP1 (ZINC TRANSPORTER 1 PRECURSOR); zinc ion transmembrane transporter
Cit.30425.1.S1_x_at	2.2	AT3G12500	AthCHIB (Arabidopsis BASIC CHITINASE); chitinase
Cit.38866.1.S1_at	2.5		
Cit.5121.1.S1_at	2.3	AT5G57685	AtGDU3 (Arabidopsis GLUTAMINE DUMPER 3)
Cit.7043.1.S1_at	3.3	AT1G17860	Trypsin and protease inhibitor family protein/Kunitz family protein

FC, fold changes; AtGID, Arabidopsis gene ID.

In addition, 103 up-regulated and 74 down-regulated Probesets are specific to the “late” stage of Las infection (Additional file 3). Interestingly, these Probesets represent some genes that belong to the categories of metabolism of carbohydrate, nitrogen and lipids, hormone IAA metabolism, response to chemical stimulus, endomembrane systems and extracellular regions. In addition, while several genes involved in cell wall property regulation (such as expansin) are up-regulated, some genes encoding transcription factors (such as MYB15/52/103) and protein kinases are down-regulated. The most striking feature is that only seven Probesets represent the very late stage-specific genes (Additional file 3). These include the genes that are most closely related to Arabidopsis C domain containing protein 71 (a transcription factor), a copper-binding family protein, a trypsin and protease inhibitor family protein/Kunitz family protein, a myosin heavy chain-related protein, two basic chitinase (CHIB) and one unknown protein encoded by At1g42430. The small number of genes belonging to this very late stage-specific category is likely due to the various experimental conditions because only 26 Probesets are commonly up- or down-regulated even in the four studies within the same very late stage of Las infection (Additional file 1). Nevertheless, as this group of genes were identified from four studies specifically at the very late stage compared to only one study for early and late stages respectively, they could be more reliable than groups of early- and late-stage-specific genes.

### Construction and characterization of gene coexpression network for citrus response to HLB

**Figure 1:**
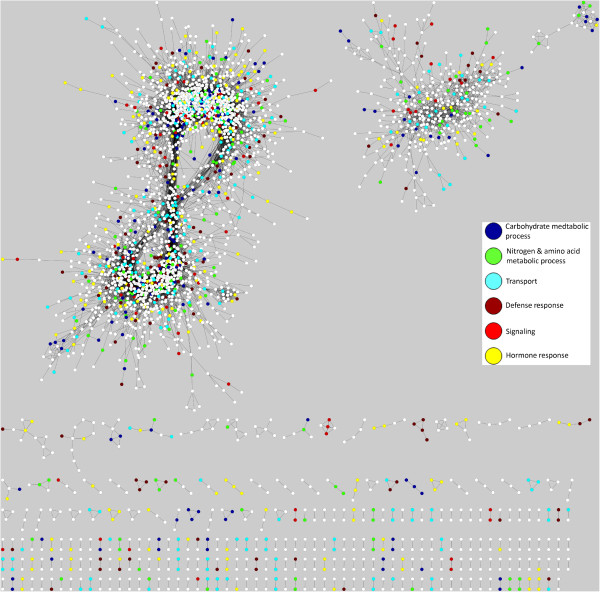
**HLB response network.** The citrus HLB response network is displayed using Cytoscape. A white circle represents a node (Probeset), and a black edge connecting the two nodes indicates an interaction (coexpression) with a Pcc value of > =0.93. Six categories of major biological processes are color-coded.

To provide a systems view of citrus host response to the HLB bacterial infection, the Pearson correlation coefficient (Pcc) method was used to infer the gene coexpression network using the four datasets reported in the four transcriptomic studies [5,10-12]. A total of 10,668 Probesets, which are present (with *P* or *M* calls) in at least two chips of the transcriptomic studies with strong expression and/or belong to the group of the HLB responsive genes (Additional file 1), were used for network analysis (see Methods). This number represents 35% of 30,173 Probesets in the citrus GeneChip. Pcc was computed between each pair of these Probesets. A Pcc threshold of 0.93 was selected, based on the overall consideration of changes in node number, edge number and network density (Additional file 4). The signed scale free R^2^ plot analysis (Additional file 4) suggests that this selection has a good scale-free topology fit, as the R^2^ value of 0.85 indicates that the topology of the HLB response network is quite similar to most biological networks. The resulting citrus gene coexpression network (called the “HLB response network” in this report) contains 3,507 nodes (Probesets) with 56,287 edges (interactions or connections) (see Figure 1 and Additional file 5).

We next determined the robustness of our network across each dataset using the cross-validation approach. We randomly left out one dataset and reconstructed the gene co-expression networks using the remaining three datasets. The resulting four networks were then compared to the network based on all four datasets in terms of network connectivity rank of each gene according to the suggestion described elsewhere [15]. There were strong, highly significant connectivity correlations (R = 0.75, 0.83, 0.83, and 0.80 respectively, each associated with a p-value of less than 2.2e-16) between the network based on all four datasets and the ones reconstructed from any combination of the three datasets. This suggests a high degree of preservation of gene co-expression patterns across the networks based on different datasets.

**Figure 2:**
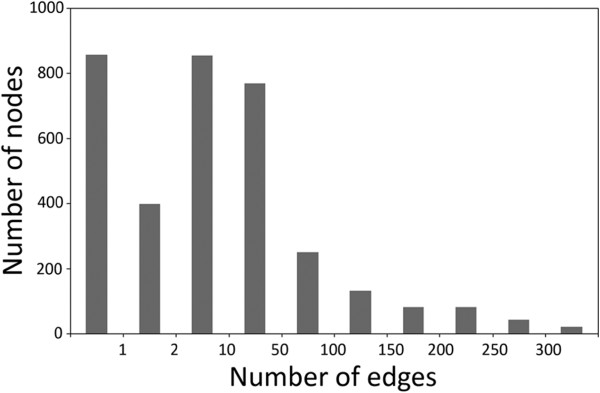
**Summary of the network characteristics.** The distribution of number of nodes (Probesets) in different ranges of edge numbers (1; 2; 3–10; 11–50, 51–100; 101–150; 151–200; 201–250; 251–300; and 300–369) in the HLB response network is presented.

We then analyzed in detail the characteristics of the HLB response network. First, the frequency distribution of edges for each node was determined. As shown in Figure 2, the network contains 860 Probesets that are orphan nodes, 400 Probesets that have only one interaction, and the majority of the nodes (2,247 Probesets or 64%) that have at least three interactions (see Figure 2) and, by following Geisler-Lee et al. [16], are called hubs in this paper. Among the 2,247 Probesets (or called hubs), the majority have 3–100 edges (1,880 Probesets or 84%), and the remaining 345 Probesets (15%) have 101–300 interactions, while only 1% (22 Probesets) have more than 300 interactions. Overall, the mean number of interactions for each Probeset is 16, with the maximum of interactions being 369 (Cit.4987.1.S1_s_at). Cit.4987.1.S1_s_at represents a gene most closely related to Arabidopsis SYP71 encoding a plant syntaxin which functions as a plasma membrane- associated protein transporter.

Second, we performed a GO enrichment analysis for the Probesets in the HLB response network. Among 30,173 Probesets, 22,775 (or 75%) have the Arabidopsis gene ID (AtGID) as their closest orthologs or homologs (Additional file 6). Therefore, these Probesets were assigned GO terms based on the most recent Arabidopsis GO assignment. The remaining Probesets were given three general GO terms: “biological process” (GO:0008150), “molecular function” (GO:0003674), and “cellular component” (GO:0005575). GO enrichment analysis using the hypergeometric statistical method with the Hochberg false discovery rate (FDR) adjustment showed that many GO terms were overrespresented in the HLB response network. Among the overrepresented GO terms (Additional file 5), the nodes belonging to the following six categories were color coded in the HLB response network (see Figure 1): carbohydrate metabolic process (213 Probesets), nitrogen and amino acid metabolic process (207), transport (311), defense response (175), hormone response (238) and signaling (218). The nodes for each of these six categories, together with the nodes belonging to some highly overrepresented GO terms such as response to stress, lipid metabolic process, cell wall and membrane part, were listed in Additional file 7. The p-values of the overrepresented GO terms were listed in Additional file 5.

We also performed a GO enrichment analysis for the hub genes. We arbitrarily divided the 2,247 hubs into two categories: minor hubs (3–99 interactions) and major hubs (> = 100 interactions) and their overrepresented GO terms were summarized in Additional file 8. The major hubs have 13 overrepresented GO terms: carbohydrate metabolic process, primary metabolic process, metabolic process, secondary metabolic process, lipid metabolic process, cellular amino acid and derivative metabolic process, cellular process, localization, transport, establishment of localization, regulation of anatomical structure size, regulation of cell size, and regulation of cellular component size. In addition to these 13 GO terms, the minor hubs have 16 additional overrepresented GO terms, such as response to stimulus (including biotic and abiotic), response to stress, regulation of biological quality and signal transduction.

### Analysis of the defense and hormone response subnetworks

Given the importance of carbon and nitrogen metabolism, transport, signaling, defense response and hormone response in the citrus response to the HLB bacterial infection and in general plant defense response, the subnetworks for these six categories were constructed by mapping the Probesets belonging to these categories into the HLB response network. The resulting edges (interactions) were listed in Additional file 7.

**Figure 3:**
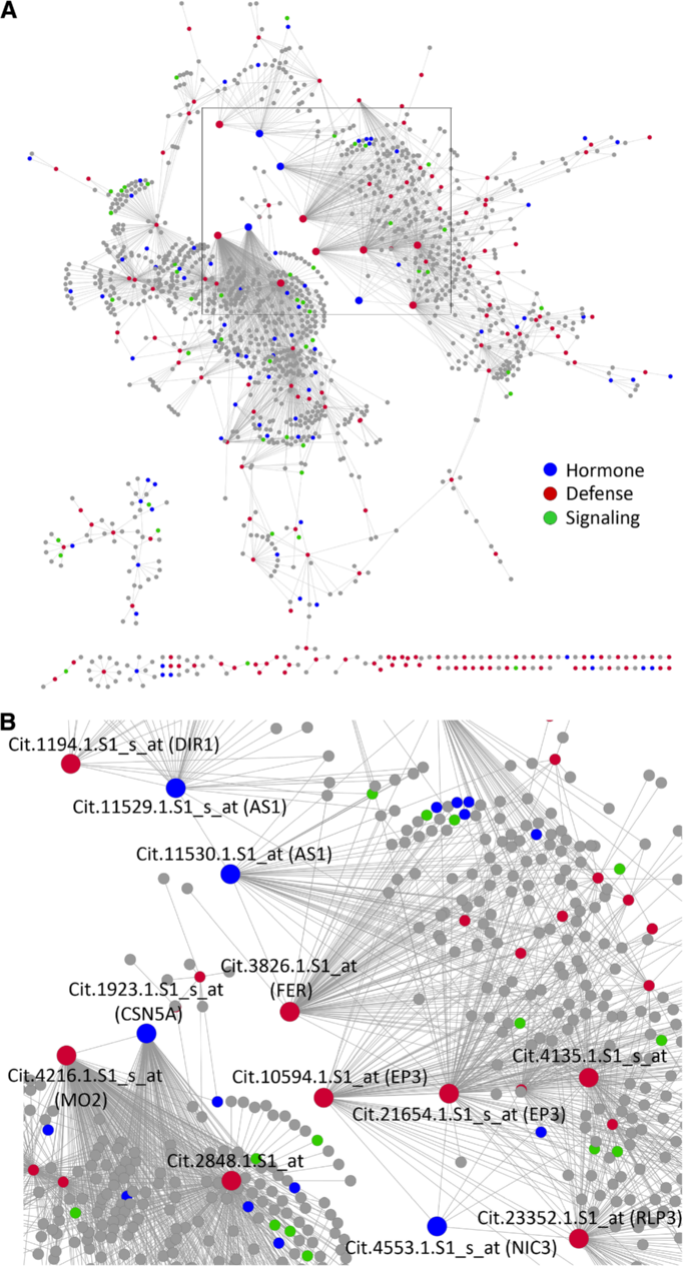
**HLB disease response subnetwork.** (**A**) The HLB defense subnetwork is displayed with three categories of major biological processes highlighted by different colors. (**B**) The close-up of the box in the subnetwork shown in Figure 3A.

We first analyzed the HLB defense subnetwork. As shown in Figure 3A, the Probesets representing defense, hormone response and signaling were color-coded. Clearly, the large hubs belong to the categories of defense and hormonal responses but not signaling. Interestingly, several of the hormone response hubs are also defense response hubs as these hubs are involved in both responses (Figure 3B). For example, the Probesets Cit.11529.1.S1_s_at and Cit.11530.1.S1_at represent a transcription factor closest to the Arabidopsis At2G37630-encoded AS1 (Asymmetric leaves 1), which is annotated as both response to fungus, virus, bacterium and salt stresses and response to hormones such as auxin, GA, SA and JA [17-19]. Interestingly, these hubs were connected to other large defense hubs such as Cit.1194.1.S1_s_at, which represents a lipid transporter closest to Arabidopsis DIR1 (Defective induced resistance 1), Cit.3826.1.S1_at, which represents a FER (FERONIA) protein kinase-like gene, and Cit.10594.1.S1_at, which represents an EP3-like chitinase gene. Cit.11529.1.S1_s_at, Cit.11530.1.S1, Cit.1194.1.S1_s_at, and Cit.3826.1.S1_at were shown to be down-regulated by the Las infections in two reports [5,11] (Additional file 1). Another example is Cit.1923.1.S1_s_at, which represents a protein degradation component similar to Arabidopsis CSN5A (COP9 Signalosome 5A) and is assigned the GO terms of both auxin response and defense response [20,21]. This hub is interconnected to at least two large defense hubs, Cit.4216.1.S1_s_at (a putative monooxygenase similar to Arabidopsis MO2) and Cit.2848.1.S1_at (a leucine-rich repeat family protein). However, there is one hormone (ABA) response hub, Cit.4553.1.S1_s_at, which represents nicotinamidase similar to Arabidopsis *NIC1* (*Nicotinamidase 1*) and is linked to at least three defense hubs. One of the connecting hub genes, Cit.23352.1.S1_at, is closest to Arabidopsis *RLP33* (*Receptor-like protein kinase 33*), and another two hubs, Cit.10594.1.S1_at and Cit.21654.1.S1_s_at, represent EP3-like chitinase genes. Although Cit.4553.1.S1_s_at itself is not HLB responsive, the above three defense hubs to which it connects were reported to be up-regulated in some of the transcriptomic studies (Additional file 1).

**Figure 4:**
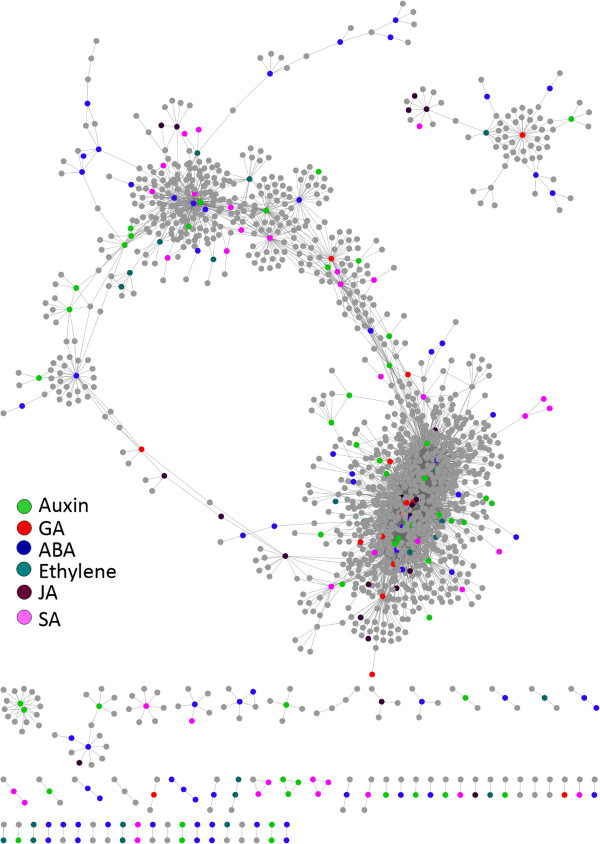
**HLB hormore subnetwork.** The HLB hormone response subnetwork is displayed, with six hormone response groups coded with distinct colors.

**Figure 5:**
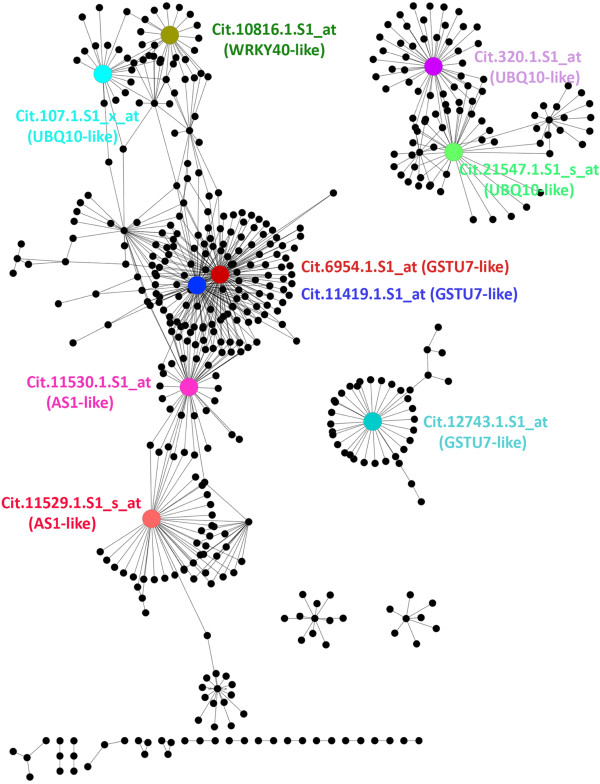
**SA response subnetwor.** The SA hormone response subnetwork is displayed. Nine large hubs are labeled with Probeset ID.

**Figure 6:**
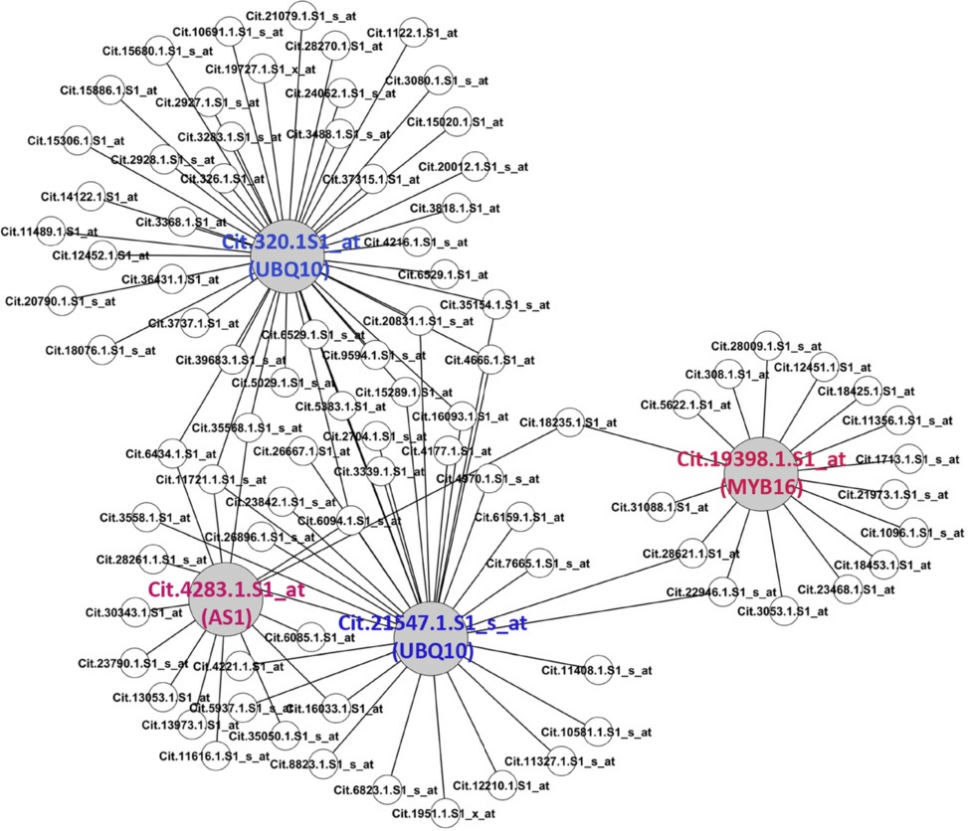
**A subset of SA response subnetwork.** A subset of the SA hormone response subnetwork with four large hubs is displayed.

The finding that the defense and hormone hubs are intertwined or overlapped (Figure 3) indicates a potentially important role for hormones in the HLB response in citrus. Also given by the increasingly clear roles for some hormones such as ethylene, ABA, JA and SA in plant defense response, we decided to analyze in more detail the hormone response subnetwork. In the HLB response network, GO terms for the response to auxin, GA, ABA, ethylene, JA and SA are overrepresented (Additional file 5) based on the hypergeometric method provided in the agriGO web tool [22] and thus the nodes for these GO terms are color-coded in the hormone response subnetwork (Figure 4) and listed in Additional file 9. It should be noted that four (auxin, GA, JA and SA) of these six overrepresented hormone GO terms are also determined to be overrepresented by using several algorithms implemented in the R package topGO which are proposed to eliminate local dependencies between GO terms [23,24]. It has been demonstrated that SA signaling is important for both local disease resistance and systemic acquired resistance [25] and a recent report showed the success in engineering the NPR1-mediated SA signaling pathway to improve citrus resistance to another destructive disease canker [26]. Therefore, we used the SA response subnetwork as an example of performing the specific hormone response network analysis. Using 49 SA response Probesets as the seed nodes, we constructed the SA response subnetwork consisting of 476 Probesets and 631 interactions (Figure 5, Additional file 9). In the SA response subnetwork, there are two major subsets, each with several large hubs. The first major subset contains transcription factors similar to Arabidopsis AS1 (Cit.11529.1.S1_s_at and Cit.11530.1.S1_at) and WRKY40 (Cit.10816.1.S1_at), protein degradation component UBQ10 (Cit.107.1.S1_x_at) and carbohydrate metabolic enzyme GSTU7 (Cit.6954.1.S1_at and Cit.11419.1.S1_at). The second major subset of the SA response subnetwork has two large hubs, both of which represent the UBQ10-like protein degradation component. A further analysis on this subset revealed that besides the two UBQ10 hubs, two other transcription factors closest to AS1 (Cit.42831.S1_at) and MYB16 (Cit.19398.1.S1_at) serve as smaller hubs linking the larger UBQ10 hubs (Figure 6). WRKY, MYB and AS1-like transcription factors have been reported to play important roles in Arabidopsis defense responses [18,19,27,28]. Ubiqutin-mediated proteasome has also been shown to be critical for plant disease resistance [29,30]. Accumulating evidence suggest that WRKY, MYB and AS1-controlled transcriptional events and ubiqutin-mediated proteasomal degradation are critical for SA signaling [18,25,31,32]. Therefore, these results strongly indicate that protein degradation and transcriptional regulation are likely critical regulatory processes involved in SA response in citrus when challenged with the Las infection.

### Analysis of the early stage HLB response subnetwork

**Figure 7:**
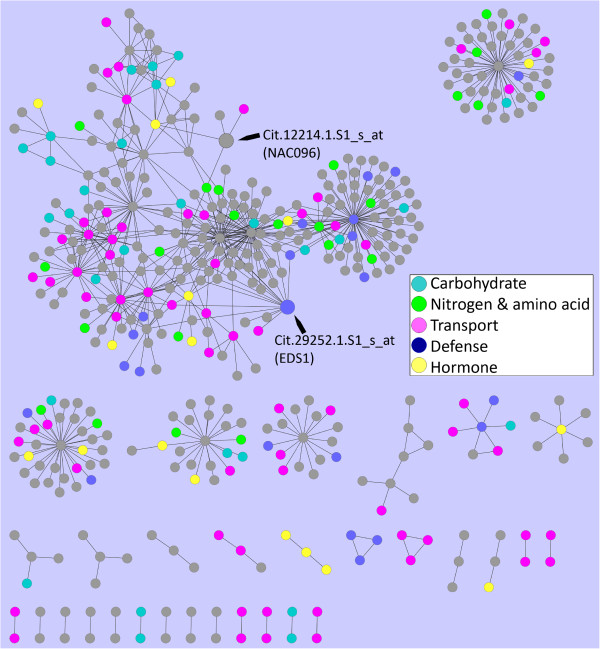
**Early stage HLB response subnetwork.** The HLB early response subnetwork is displayed. Nodes belonging to five categories of major biological processes are coded with distinct colors. The arrows indicate the two nodes (Probesets) that are discussed in the text.

At early stage, the HLB bacterium could rarely be detected, nor any HLB symptom observed, but the response to HLB in citrus could occur early at least at the transcriptional level. Therefore, we decided to analyze the subnetwork for the early-stage HLB responsive genes. A total of 222 Probesets, including 158 up-regulated and 62 down-regulated Probesets (Table 1, Additional file 10), were used as the seed nodes to map the HLB response network, resulting in the HLB early response subnetwork (Figure 7). This subnetwork based on the first degree neighbors of these seed nodes contains 461 Probesets and 683 interactions (Additional file 10). Among those Probesets, 29 are involved in carbohydrate metabolic process, 23 in nitrogen and amino acid metabolic process, 67 in transport, 27 in defense response, 24 in signaling and 24 in hormone response. GO enrichment analysis shows that carbohydrate metabolic process (p-value = 3.6E-4), transport (p-value = 4.1E-10) and defense (p-value = 6.3E-3) are overrepresented. Although the hormone response category is not overrepresented, JA response consisting 10 Probesets is overrepresented with a p-value of 0.01). Therefore, our analysis of the early stage subnetwork indicates that even at this stage (5–9 weeks after inoculation), several important biological processes have been activated or inactivated. In the HLB early response subnetwork, there is only one subset that has several large hubs, while all other small subsets have interactions that are not connected further (Figure 7). To provide further detail of the early stage response in citrus, we analyzed the two nodes in the large subset of this subnetwork, Cit.29252.1.S1_s_at, and Cit.12214.1.S1_s_at.

**Figure 8:**
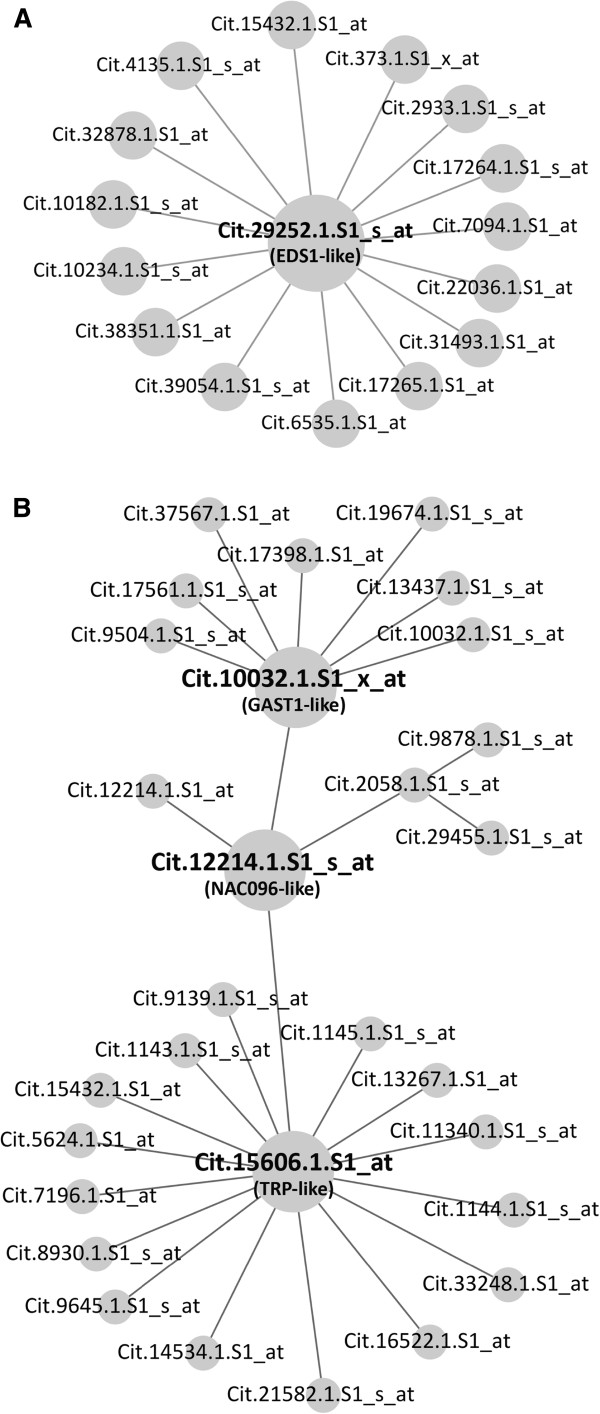
**Early stage HLB response subnetworks based on two representative genes.** Two gene-specific subnetworks are displayed with all Probesets labeled. (**A**) A subset of early stage HLB response subnetwork centered on the hub Cit.29252.1.S1_s_at. Because many second degree interactions exist for most of the first degree nodes connecting to Cit.29252.1.S1_s_at, only the first degree interaction-based subnetwork is presented. (**B**) The NAC096 transcription factor subnetwork is displayed.

Cit.29252.1.S1_s_at represents a triacylglycerol lipase gene most closely related to Arabidopsis *EDS1* (Enhanced disease susceptibility 1) [33]. Extracting this *EDS1*-like gene from the HLB early response subnetwork (Figure 7) shows that *EDS1* interacts with 15 Probesets (Figure 8A). Among these Probesets, one Probeset (Cit.38351.1.S1_at representing a MATE efflux family protein) has interactions with only two other Probesets, five Probesets (Cit.6535.1.S1_at, Cit.15432.1.S1_at, Cit.10234.1.S1_s_at, Cit.4135.1.S1_s_at, and Cit.2933.1.S1_s_at) form the large hubs each with 50–113 interactions, and nine other Probesets form the medium-size hubs with 11–44 interactions. The fact that Cit.29252.1.S1_at connects with the five large hubs indicates a potentially critical role in citrus response to the HLB bacterial infection. Cit.6535.1.S1_at represents a carbohydrate transmembrane transporter or phosphate transmembrane transporter, Cit.10234.1.S1_s_at is closely related to CB5-E (Cytochrome B5 isoform E) involved in heme binding, Cit.4135.1.S1_s_at represents a putative CC-NBS-LRR class disease resistance protein, and Cit.2933.1.S1_s_at is very similar to Arabidopsis HMGB1 (High mobility group 1) involved in transcriptional control through chromatin remodeling. In addition, some of the medium-size hubs that interact with the *EDS1*-like gene play important roles in protein modifications or lipid metabolism. For example, Cit.39054.1.S1_s_at is closely related to Arabidopsis *SAG101* (*Senescence-associated gene 101*) which encodes a carboxylesterase or triacylglycerol lipase and has been shown to physically interact with EDS1 [34], and Cit.373.1.S1_x_at is similar to UBQ10 (POLYUBIQUITIN 10). The *EDS1*-like citrus gene was up-regulated at the early stage (2.0-fold difference) and at the very late stage (6.5-fold difference; Additional file 1) in only one of the four studies [11], and most of 15 hub genes that interact with the citrus *EDS1*-like gene were also up-regulated by the Las infection in some of the studies with the exception of Cit.373.1.S1_x_at (UBQ10), Cit.39054.1.S1_s_at (SAG101) and Cit.10182.1.S1_s_at (an aldo/keto reductase family protein) (see Additional file 1). Therefore, the finding that so many HLB responsive hub genes in citrus connect to *EDS1*, which is critical for disease resistance in Arabidopsis and other plants [33,35,36], indicates that EDS1-mediated defense response mechanism might be important in citrus response to the HLB bacterial infection at least at early stage.

Cit.12214.1.S1_s_at represents a transcription factor most closely related to Arabidopsis NAC096. Mapping this Probeset as the seed node to the HLB response network with the second degree neighbors resulted in an NAC096 subnetwork (Figure 8B). Two medium-size hubs were identified in this subnetwork: Cit.10032.1.S1_x_at and Cit.15606.1.S1_at, both of which were up-regulated transcriptionally by the Las infection (Additional file 1). Cit.10032.1.S1_x_at represents a GA-responsive GAST1 homolog and has been reported to be responsive to other hormones such as BR and ABA [37,38]. Cit.15606.1.S1_at has interactions with 15 Probesets and is closely related to At1g80130 which encodes Arabidopsis tetratricopeptide repeat (TPR)-like superfamily protein and is responsive to oxidative stress [39]. Given that both GA response and oxidative stress response have been implicated an important role in a relatively resistant variety US-897 in response to the Las infection at the very late stage [12], our preliminary analysis of the NAC096 subnetwork supports that transcriptional control involving hormone response and oxidative stress response might also be important even at the early stage of the HLB bacterial attack.

### Subnetwork analysis reveals transport process as a key component in the HLB response core subnetwork

**Figure 9:**
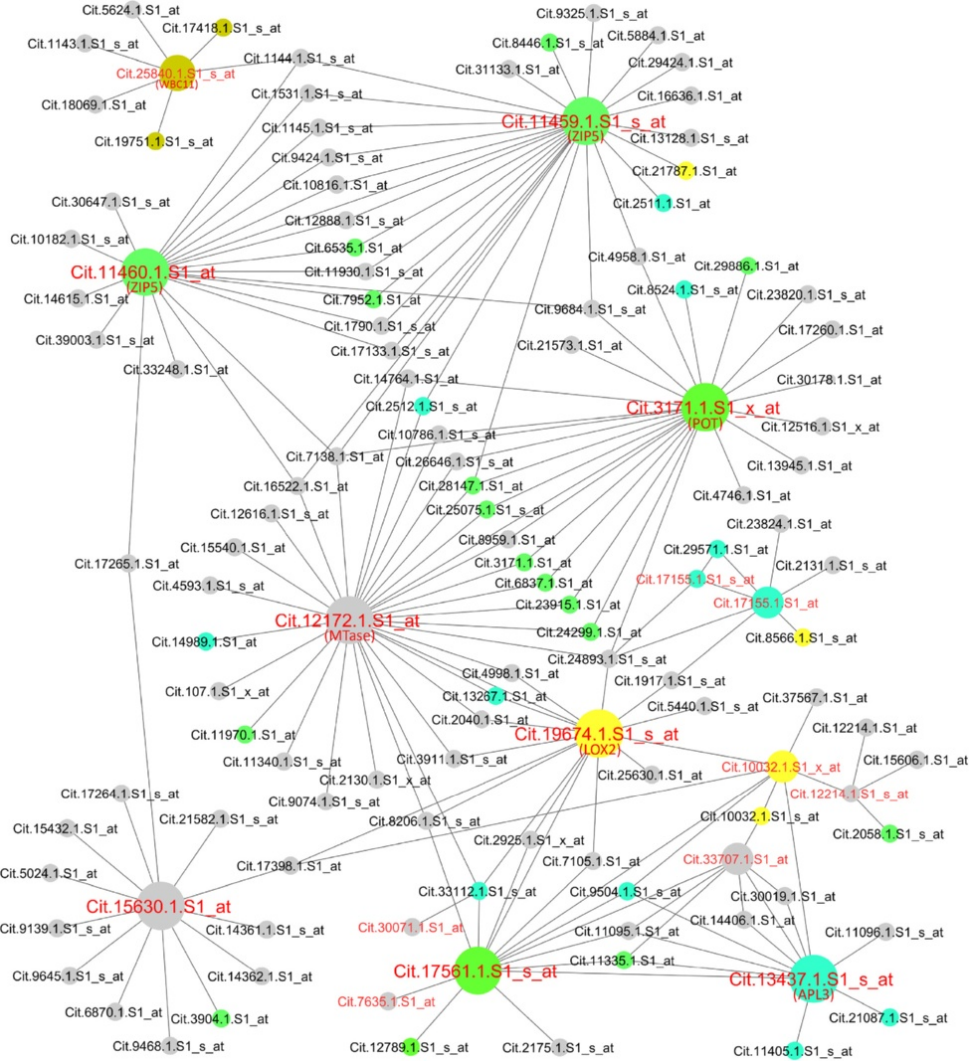
**HLB response core subnetwork.** The HLB response core subnetwork is constructed using the 21 commonly up-regulated Probesets (see Table 2) as the seed nodes (highlighted in red). First degree interactions are shown. Green nodes indicate “transport”, yellow nodes represent “hormone response”, and blue nodes indicate “carbohydrate metabolic process”. Nodes overlapped in the categories of “transport” and “hormone response” exhibit a mixed color of green and yellow.

It is likely that the commonly up-regulated genes can define a default or core response pathway for citrus plants to resist the attack by the HLB bacteria; we therefore attempted to address whether there is a common subnetwork that could be affected by HLB. We mapped 21 commonly up-regulated Probesets into the HLB response network, resulting in the formation of the HLB core subnetwork (Figure 9). This subnetwork based on the first degree neighbors contains 123 Probesets and 181 interactions (Additional file 11). The hub gene analysis shows that the subnetwork has eight large hubs, all of which were up-regulated, and four small hubs (Figure 9). Among six categories of biological processes analyzed in the HLB response network, transport and carbohydrate metabolic process were overpresented in this core subnetwork (p-value = 1.0E-05 and 1.3E-4, respectively). The Probesets belonging to the three categories, carbohydrate metabolic process, transport and hormone response, were then color-coded in this subnetwork (Figure 9). The largest category of the genes in the subnetwork belongs to transport process, with a total of 22 Probesets (representing 18% of the nodes in the core subnetwork versus 6% in the HLB response network). Among these Probesets, four form the large hubs: Cit.11459.1.S1_s_at, Cit.11460.1.S1_at, Cit.3171.1.S1_x_at, and Cit.17561.1.S1_s_at. Given the importance of hub genes in the biological networks and overrepresentation of transport in the subnetwork, we propose that transport process is a key component in the HLB response core subnetwork.

There are 13 Probesets grouped into the category of carbohydrate metabolic process and 11 Probesets that belong to the hormone response category. For the category of carbohydrate metabolic process, Cit.13437.1.S1_s_at forms a larger hub with 11 interactions, and Cit.17155.1.S1_at forms a smaller hub with seven interactions. Cit.13437.1.S1_s_at represents a citrus gene similar to Arabidopsis *APL3* encoding a glucose-1-phosphate adenylyltransferase. Cit.17155.1.S1_at represents a gene closely related to BGLU11 (Beta-glucosidase 11) hydrolysis of O-glycosyl compounds. For the hormone response category, Cit.19674.1.S1_s_at forms a larger hub with 15 interactions, and Cit.10032.1.S1_x_at and Cit.25840.1.S1_s_at form smaller hubs with seven and six interactions respectively. As described above, Cit.19674.1.S1_s_at represents a gene closely related to *LOX2* encoding a lipoxygenase and exhibiting response to JA. In Arabidopsis, LOX2 has also been shown to be involved in JA biosynthesis in response to wounding [40,41] and recently in disease development [42]. As described previously, Cit.10032.1.S1_x_at represents a GA-responsive GAST1 homolog and is connected to the NAC096 transcription factor subnetwork in the HLB early response subnetwork (Figures 7 and 8B). Interestingly, Cit.25840.1.S1_s_at represents a gene very similar to Arabidopsis *WBC11* (White-brown complex homologous protein 11) which encodes an ATPase coupled to transmembrane movement of substances or fatty acid transporter [43]. This small hub is responsive to ABA and salt stress but is also involved in fatty acid transport, implying a potential role for hormone signaling in the control of transport process.

The remaining two large hubs in the HLB response core subnetwork are formed by Cit.12172.1.S1_s_at and Cit.15630.1.S1_at. Cit.12172.1.S1_s_at represents a putative *O*-methyltransferase family 2 protein most closely related to the protein encoded by At4g35160. At4g35160 is only annotated as a general GO term “methylation”, and predicted to contain a winged helix-turn-helix transcription repressor DNA-binding domain without any functional implication. This hub includes 31 interactions, and most of the interactions are with the Probesets related to transport process. Cit.15630.1.S1_at represents a gene closest to At4g33040 which encodes a glutaredoxin family protein. It connects to a transportor hub through Cit. 17265.1.S1_at (a glycine-rich protein without any specific functional annotation) and the two hormone response hubs through Cit.17398.1.S1_at (which represents e gene encoding an unknown protein). In Arabidopsis, At4g33040-encoded glutaredoxin family protein is annotated as a protein involved in cell redox homeostasis, which could have a potential role in response to oxidation stress.

### Analysis of a phloem protein subnetwork implicates a potential role for zinc transport in the citrus HLB defense response

**Figure 10:**
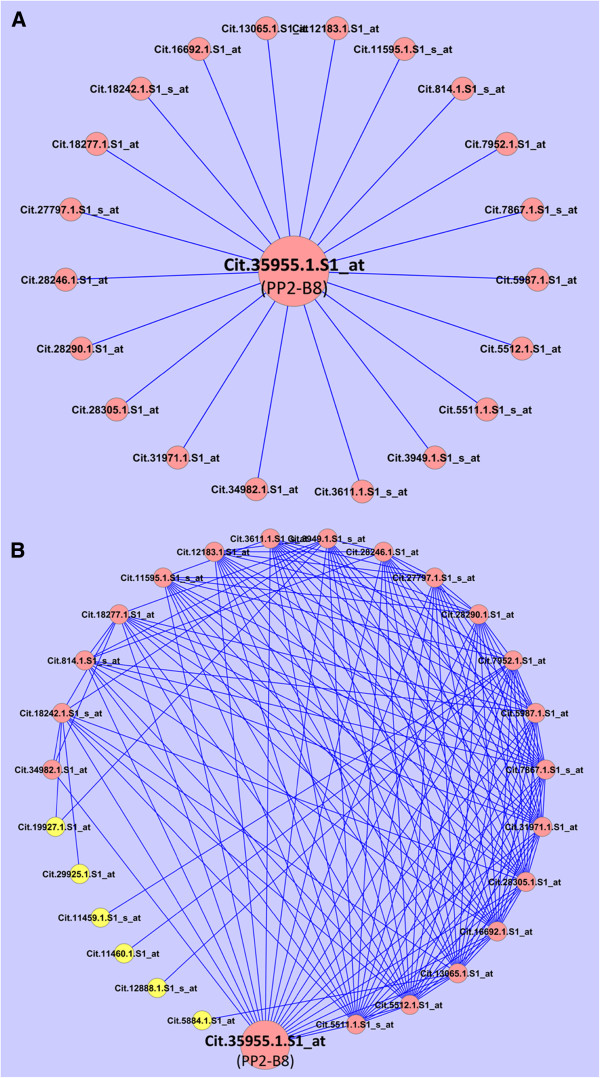
**Phloem protein (PP2) subnetwork.** The subnetworks for the PP2, based on first (**A**) and second (**B**) degree neighbors of Cit.35955.1.S1_at. Six of the Probesets belonging to the second degree interactions (**B**) are labeled with yellow.

Given the potential importance of phloem protein 2 (PP2)-type lectin in phloem morphogenesis in particular the formation of sieve plug [5,6,44], PP2-like protein genes in citrus were used as an example to further illustrate the application of the HLB response network. A survey of ten PP2-like genes present in the citrus GeneChip showed that four of the PP2-like genes were up-regulated and one down-regulated (Additional file 12). Although their expression pattern was quite different, one gene represented by the Probeset Cit.35955.1.S1_at was dramatically up-regulated at late stage (44.6-fold difference) and very late stage (42–506 fold differences) in all of the four reports [5,10-12] except for the relatively resistant variety US-897 which did not exhibit any activation at very late stage (Additional file 1). This gene is closely related to Arabidopsis PP2-B8. This Probeset and the other, Cit.3272.1.S1_s_a, are present in the HLB response network. The latter one represents a PP2-A15-like gene but expression of this gene was not affected by HLB in any of the four reports and it only connects with three genes in the HLB response network (Additional file 12). The lack of activation of Cit.35955.1.S1_at by the Las infection at the early stage might be due to that the HLB symptom has not been fully developed yet. When the PP2-B8 subnetwork was constructed, we found that this gene connects with 20 Probesets (Figure 10A) which are interconnected frequently between each other (Figure 10B). Furthermore, seven of the 20 first degree interacting Probesets represent the genes involved in transport, and three of these genes are predicted to encode zinc transporters (Additional file 12). In addition, four Probesets represent genes encoding zinc-binding proteins. Given that HLB disease symptom was initially thought to be related to zinc deficiency [1,2], our network analysis approach provides an intriguing possibility for zinc transporters or zinc-binding proteins to function in citrus response to the HLB bacterial infection.

## Discussion

The transcriptomes in citrus in response to the HLB bacterial infection have been well documented in four previous reports [5,10-12], but the information regarding the interactions between the differentially expressed genes is lacking. Through the combination of transcriptome comparative study and gene coexpression network analysis, we have provided for the first time a systems view of how the citrus host plant exerts a genome-wide response to the HLB bacterial infection.

First, we have constructed an HLB response network involving 3,507 Probesets with 56,287 interactions. Using the transcriptome datasets and orthology-based or experimentally verified protein-protein interaction datasets, gene-gene interactions or interactomes have been constructed in the model plants including Arabidopsis and rice (for example, [16,45-52]) and occasionally in non-model plants such as soybean [53] and barley [54]. However, there was no report on gene-gene interaction networks in citrus prior to our work. We used the Pcc method to construct a gene coexpression network in citrus, with a focus on the HLB response mechanism. The citrus gene coexpression network will be very useful for the citrus researchers to visualize the subnetworks specific for certain biological processes (such as carbohydrate metabolic process or signaling not analyzed in detail in this report), or to search some potential gene-gene interactions for certain genes or a group of genes in the future. The Citrus Gene Interaction Networks (CitGIN) database has been constructed and made available to the research community to query through the Internet (http://xt.cric.cn/cgr/CitGIN.php).

Second, our analysis of the defense subnetwork has shown that many defense hubs and hormone hubs are intertwined or overlapped. Although the roles of hormone and defense response genes have been discussed in the four previous reports [5,10-12], our network analysis further indicates that hormone response is interconnected to defense response in citrus when challenged by the HLB bacteria. This may lead to the development of integrating hormone and disease response pathways as a potentially more effective genetic means to improve the citrus resistance to HLB.

Third, our comparative studies of transcriptomes have led to the identification of subsets of commonly up-regulated and stage-specific HLB responsive genes. In contrast to those four GeneChip reports where various statistical methods and fold-change cutoffs were used, we used the same procedure for the analysis of all of the transcriptome datasets. Furthermore, by mapping the subset of commonly up-regulated genes into the HLB response network, we have found that the genes belonging to the categories of carbohydrate metabolic process, transport and hormone response are positioned as the large hubs in the HLB response core subnetwork. This indicates that these three processes constitute a core subnetwork for the citrus host response to the HLB bacterial infection. In addition, we propose that transport is a key component in this HLB response core subnetwork.

Fourth, using PP2 gene as an example of applying the HLB response network, our subnetwork analysis provides an intriguing possibility for the zinc transporter or zinc binding proteins to act with PP2 protein in response to the HLB bacterial infection. PP2 proteins belong to a large gene family in higher plants. However, they have not been assigned a specific biological process, and thus their biological function remains unknown. They are predicted to bind carbohydrates and have been implicated a role in the formation of sieve plug or replacement phloem [5,10,12,44]. Some of the PP2 genes from other organisms such as melon, cucumber and Arabidopsis are specifically or preferentially expressed in companion cells but their protein products are translocated in sieve elements [44]. This indicates a role for PP2 proteins not only in intracellular signaling but also in long distance intercellular communication [44]. Recent evidence show that PP2-type proteins might be involved in aphid-mediated virus transmission in cucumber [55] and overexpression of *PP2-A1* in Arabidopsis increased resistance to a phloem-feeding insect [56], indicating a possibly more active role for PP2 family proteins in defense response in plants. In citrus, the Probeset Cit.35955.1.S1_at, which is closely related to Arabidopsis PP2-B8, was dramatically up-regulated at late stage and very late stages. The most surprising feature of the PP2-B8 subnetwork is that the 20 Probesets, which are the first degree neighbors of Cit.35955.1.S1_at, are interconnected frequently between each other. This indicates that these genes might be regulated by the precise coordination of various signaling pathways through transcription factors, chromatin modification or remodeling proteins or other factors. Furthermore, seven of the 20 interacting Probesets encode proteins involved in transport, consistent with our proposal that transport is a key component in the HLB response core subnetwork. In addition, three of the seven transporters are predicted to transport zinc, and the PP2 subnetwork also contains four Probesets which represent the genes encoding zinc-binding proteins. Intriguingly, HLB disease symptom was initially thought to be related to zinc deficiency and the zinc transport system is required for virulence in other organisms [1,2], and therefore the PP2 subnetwork analysis indicates that zinc transporters or zinc binding proteins may have a potentially important role for citrus to respond to the HLB bacterial infection. Taken together, our analysis using the HLB response network can lead to an intriguing but testable hypothesis (which cannot be done without the network analysis) regarding the role of PP2 proteins and zinc transport system or zinc binding proteins in citrus HLB defense response.

It should be noted that there are some potential limitations in our network study. The first one is GO enrichment analysis. The agriGO web tool, which is based on the hypergeometric method and used in this work, does not take into account the local dependency of GO terms. Using the four algorithms provided in the topGO R package which are proposed to eliminate local dependencies, we have found that four of the six hormone GO terms determined to be overrepresented by agriGO are also overrepresented, while the two other hormones (ABA and ethylene) have their child GO terms being truly overrepresented. Therefore, different algorithms or statistical methods in GO enrichment analysis will probably lead to some differences in terms of the overrepresented GO terms for the nodes in the HLB response network.

The second limitation is due to the small sample size. Computational prediction of gene-gene interactions usually requires large sample size; however relatively small number of samples were recently used to construct gene coexpression networks specific to certain aspects of biology (such as [50,52,57]). In our analysis, we used the transcriptome datasets described in four previous reports [5,10-12]. Among these, only one study is available for early stage and late stage respectively, while there are four studies for very late stage (Table 1). We have found that only a small number (222) of significantly regulated Probesets can be identified for early stage, while almost 600 and 2,000–4,000 differentially expressed Probesets can be found for late and very late stages respectively. The variation in the number of differentially expressed genes at different stages could be caused by the difference in experimental conditions given that different ages and varieties of trees and different sources of inoculants were used in different years in those four reports. However, this variation might lead to some sort of bias towards the very late stage genes. To minimize the possibility that the interactions we have detected were the result of random events due to the small sample size, we have selected a high Pcc cutoff value which has led us to believe that the interactions are more likely statistically significant rather than by random and that the topology of the HLB response network is quite similar to most biological networks. Furthermore, the cross-validation result shows a high degree of preservation of gene coexpression patterns, suggesting that the HLB response network is at least moderately robust and biologically relevant. Therefore, despite some limitations due to the small sample size and the experimental variations, the network reported here should be quite useful for the citrus research community and have provided some novel insights into the citrus HLB defense mechanisms. When larger scale transcriptome datasets become available in the future, similar network analysis will provide a comprehensive picture of the gene networks in citrus.

The most daunting challenge in the citrus postgenomic era remains how to identify the best candidate genes for functional dissection of the HLB response mechanism and for genetic modification with an ultimate goal of improving the HLB resistance in citrus. Genetic variations of HLB susceptibility [9] clearly shows the potential towards dissection of genetic mechanisms of HLB resistance, but understanding the inheritance patterns and subsequently cloning the disease genes requires a long term effort because of long juvenile phase and complex reproductive biology for citrus. Recent developments have shed some lights into the identification of key hub genes as candidate regulatory genes. For example, a seed germination study found that 22–50% of the Arabidopsis hub genes identified from the seed germination network actually have physiological functions in the control of seed germination [49]. Therefore, the hub genes identified in this report may potentially be the first batch of candidates for the functional test in HLB resistance in citrus.

## Conclusions

Through integration of transcriptome comparison and gene coexpression network analysis, we have provided novel insights into the mechanism by which citrus host plants respond to the HLB bacterial infection. Specifically, several biological processes are important in the citrus HLB response network, including carbohydrate metabolic process, nitrogen and amino acid metabolic process, transport, defense response, signaling and hormone response. Furthermore, our results have led us to propose that transport is a key component in the HLB response core subnetwork. This systems view of citrus response to the *Ca.* Liberibacter spp. infection will be a critical first step towards dissecting the genetic mechanisms of HLB response and ultimately improving HLB resistance in citrus.

## Methods

### Data collection and preprocessing

Raw data for citrus Affymetrix GeneChip analysis published by Fan et al. [11] (GSE29633) and Albrecht and Bowman [12] (GSE30502) were downloaded from NCBI. Raw data published in [10] (GSE33459) and [5] were kindly provided by Drs. Bowman and Wang, respectively. These *.cel* files were read into R and preprocessed using “rma” function [58] and normalized using the “normalize.quantiles.robust” function. After quantile normalization, Probesets with an absent (A) call were removed using the “pma” function. Probesets with the calls of present (*P*) or marginal (*M*) in at least two samples in each of the four reports above were included in the analysis. All of the statistical analysis and gene expression network construction were performed in the R environment.

### Analysis of significantly regulated genes

The adjusted local pooled error (LPE) method [13] was used to identify differentially expressed transcripts, as this method has been shown to provide high power in analyzing microarray data with small sample size. A gene was called statistically significant if its permutation-based false discovery rate (FDR) p-value was smaller than 0.05 and at least a two-fold change was observed.

### Network construction and visualization

For computational reasons, up to 10,000 of the Probesets with highest expression levels were selected from each of the datasets described in the four reports [5,10-12]. The HLB responsive genes identified in this study (described above) were then added to this list and duplicated ones were removed, resulting in a total of 10,668 common Probesets for each of the four datasets. Gene coexpression network was constructed from the preprocessed files using R package “weighted correlation network analysis” [59]. Following the protocol for constructing gene co-expression network using multiple datasets [15], we first calculated Pearson correlation matrix for each dataset. We then obtained an overall weighted correlation matrix based on the number of samples used in that dataset. The weight for each correlation matrix was defined as $w_{i}=\frac{\sqrt{n_{i}}+\sqrt{n_{\text{max}}}}{\sum_{i}n_{i}+s\sqrt{n_{\text{max}}}}$, where *n*_*i*_ was the number of samples for *i*th dataset, *n*_*max*_ was the maximum number of samples in all datasets, and *s* was the number of datasets used. Two nodes were determined to be connected if the absolute value of the Pearson correlation coefficient (Pcc) exceeded 0.93. The threshold of 0.93 was selected such that it gave the best overall fit to each dataset based on the criteria such as the scale-free topology model fitting index, mean network connectivity, and network density. Cytoscape v2.8.2 [60] was used to visualize the networks and Photoshop was used to edit the images.

### GO analysis and Arabidopsis orthology prediction

Because of the lack of citrus genome annotation for the Probesets in the Affymetrix chip, the Probesets were used for all analysis. They were annotated using Arabidopsis orthologs or homologs. The Probesets were annotated by searching against the Arabidopsis genome using the tool provided in HarvEST database (http://harvest-web.org). GO terms were assigned to the citrus Probesets based on their corresponding Arabidopsis gene ID (AtGID). For those without AtGID, general GO terms were assigned: “biological process” (GO:0008150), “molecular function” (GO:0003674), and “cellular component” (GO:0005575). GO enrichment analysis was performed using the hypergeometric statistical method with Hochberg FDR adjustment in the AgriCO website (http://bioinfo.cau.edu.cn/agriGO/analysis.php) as described elsewhere [22].

## Abbreviations

HLB: Huanglongbing; Las: *Candidatus* Liberibacter asiaticus; Pcc: Pearson correlation coefficient; GO: Gene Ontology.

## Competing interests

The authors declare that they have no competing interests.

## Authors’ contributions

ZLZ collected the data and carried out biological analysis and interpretation. YZ performed statistical analysis. ZLZ and YZ wrote the manuscript. Both authors approved the manuscript.

## Supplementary Material

Additional file 1 A list of HLB responsive genes reported in this analysis. Click here for file

Additional file 2 Number of overlapped genes in various HLB studies. Click here for file

Additional file 3 A list of stage-specific HLB responsive genes. Click here for file

Additional file 4 PCC cutoff selection and network topology characteristics. Click here for file

Additional file 5 A list of coexpressed genes and their interactions and the GO enrichment analysis result. Click here for file

Additional file 6 CitProbeID_AtGID annotation_GO terms. Click here for file

Additional file 7 A list of nodes and edges in six major subnetworks and nodes in some highly overrepresented GO terms. Click here for file

Additional file 8 GO enrichment analysis of hub genes in the HLB response network. Click here for file

Additional file 9 Lists of nodes and edges in the hormone subnetworks. Click here for file

Additional file 10 A list of nodes and edges in the early-stage HLB response subnetwork. Click here for file

Additional file 11 A list of nodes and edges in the HLB response core subnetwork. Click here for file

Additional file 12 A list of PP2-like genes in the citrus GeneChip and the genes interacting with Cit.35955.1.S1_at. Click here for file
