# Supplementary figures and images for: Transcriptome comparison and gene coexpression network analysis provide a systems view of citrus response to ‘Candidatus Liberibacter asiaticus’ infection
Source: BMC Genomics. 2013 Jan 16;14:27. doi: 10.1186/1471-2164-14-27 (PMC3577516; doi:10.1186/1471-2164-14-27)

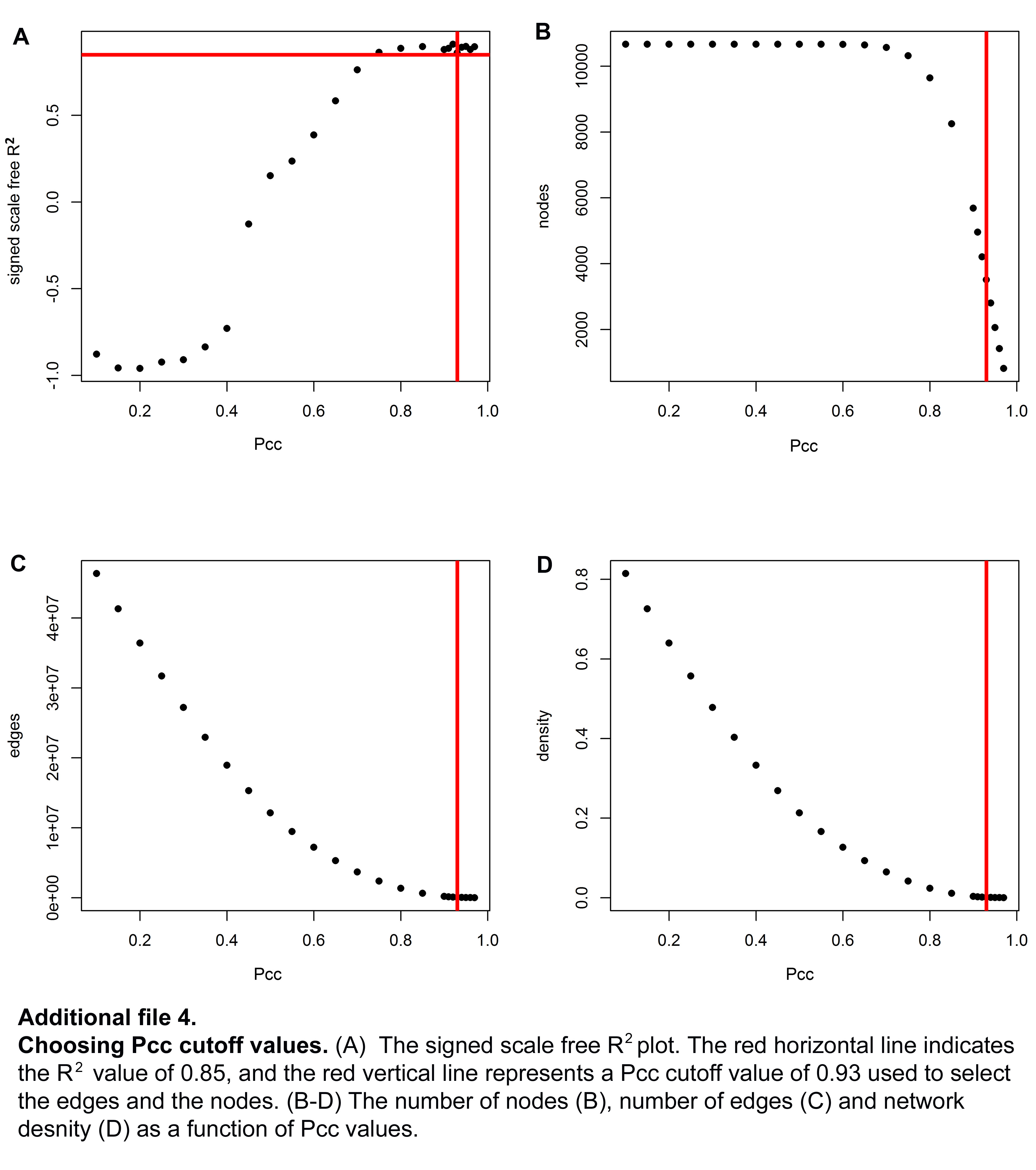

Supplement: Additional file 4 — PCC cutoff selection and network topology characteristics. [file 1471-2164-14-27-S4.jpeg]
